# Functional/activity network (FAN) analysis of gene-phenotype connectivity liaised by grape polyphenol resveratrol

**DOI:** 10.18632/oncotarget.9578

**Published:** 2016-05-24

**Authors:** Tze-chen Hsieh, Sheng-Tang Wu, Dylan John Bennett, Barbara B. Doonan, Erxi Wu, Joseph M. Wu

**Affiliations:** ^1^ Department of Biochemistry and Molecular Biology, New York Medical College, Valhalla, New York 10595, U.S.A; ^2^ Division of Urology, Department of Surgery, Tri-Service General Hospital, National Defense Medical Center, Taipei, Taiwan, ROC; ^3^ Department of Neurosurgery, Baylor Scott and White Health, Temple, Texas, 76508, U.S.A; ^4^ Department of Surgery, Texas A&M College of Medicine, Temple, Texas 76504, U.S.A; ^5^ Department of Pharmaceutical Sciences, Texas A&M Health Science Center, College Station, Texas 77843, U.S.A

**Keywords:** resveratrol, functional/activity network analysis, connectivity, cancer

## Abstract

Resveratrol is a polyphenol that has witnessed an unprecedented yearly growth in PubMed citations since the late 1990s. Based on the diversity of cellular processes and diseases resveratrol reportedly affects and benefits, it is likely that the interest in resveratrol will continue, although uncertainty regarding its mechanism in different biological systems remains.

We hypothesize that insights on disease-modulatory activities of resveratrol might be gleaned by systematically dissecting the publicly available published data on chemicals and drugs. In this study, we tested our hypothesis by querying DTome (Drug-Target Interactome), a web-based tool containing data compiled from open-source databases including DrugBank, PharmGSK, and Protein Interaction Network Analysis (PINA). Four direct protein targets (DPT) and 219 DPT-associated genes were identified for resveratrol. The DPT-associated genes were scrutinized by WebGestalt (WEB-based Gene SeT Analysis Toolkit). This enrichment analysis resulted in 10 identified KEGG (Kyoto Encyclopedia of Genes and Genomes) pathways. Refined analysis of KEGG pathways showed that 2 — one linked to p53 and a second to prostate cancer — have functional connectivity to resveratrol and its four direct protein targets. These results suggest that a functional activity network (FAN) approach may be considered as a new paradigm for guiding future studies of resveratrol. FAN analysis resembles a BioGPS, with capability for mapping a Web-based scientific track that can productively and cost effectively connect resveratrol to its primary and secondary target proteins and to its biological functions.

## INTRODUCTION

Resveratrol is a stilbenoid present abundantly in red wine, red grape skin, peanuts and several other food items and drinks consumed daily. Interest in resveratrol increased greatly following the report by Jang et al. in 1997 showing that it displays chemopreventive activities as evidenced by the inhibition of carcinogenesis using *in vitro* experiments and animal model studies [1]. Resveratrol has also been shown to have beneficial effects on coronary heart diseases, the aging process, pathological inflammation, and ischemic and chemically induced injuries. Resveratrol-mediated bioactivities are of significant public health interest; accordingly, research focus in recent years has been directed to the understanding of its mechanisms and identification of its primary (direct) and secondary (indirect) targets [2–11]. What approach is most appropriate and relevant for the systematic dissection of the rich published research data on resveratrol to generate insightful, definitive and predictive leads, and not merely random clues typically associated with a hit-and-miss strategy? Can this approach also compare experimental findings from the same or different laboratories, insofar as the functional interpretation of changes in large gene sets for possible extrapolation into common or unique underlying biological themes?

In recent years, the successful launch and implementation of numerous multi-center genomics studies, encompassing the use of microarrays, proteomics, and other high-throughput screening assays often produce hundreds to thousands of intriguing gene hits which can present formidable challenges for integration, phenotypically or thematically. A possible solution to this analytical obstacle in data mining may lie in the use of the network-based approach — a simple yet effective method that has found application in the analysis of massive data obtained from numerous human disease studies designed to explore and connect the relationship existing between a drug and its targets and interacting proteins, for association with disease [12–15]. DTome is a web-based instrument that allows for the search and mining of existing drug-target information [16]. DTome could provide insight and understanding of the molecular mechanisms of drug action as well as a focused scope and direction for future research. Whether DTome can reveal authentic or teaser leads and generate rational, clinically relevant hypotheses for advancing our knowledge on chemicals or dietary agents with chemopreventive potential remains to be established.

In this study, we introduce a FAN analysis capable of effectively analyzing and prioritizing candidate genes quickly for linkage to experimentally verifiable grouped pathways through the deployment of Web-accessible, open portal databases. As test of principle, we applied DTome to the widely studied polyphenol resveratrol. We found that 3 of its 4 identified direct protein targets are linked to the tumor suppressor gene p53. When the readout targets and their associated genes were further analyzed for functionality through pathway enrichment analysis, the p53 pathway was identified as one of top 10 enriched KEGG pathways containing 8 resveratrol-associated genes. We propose that the DTome-directed FAN analysis is a new paradigm for analyzing resveratrol:p53 interplay; the flagged genes involved in resveratrol-mediated p53 control can be experimentally verified and new insights on the biological context of activation/restoration of p53 function by resveratrol might be illuminated. The approach has implications for understanding the prevention of cancer by resveratrol and can aid in the treatment of malignant tumors harboring the dysfunctional p53.

## RESULTS

### Characterization of bioactivities of resveratrol using DTome and visualization of resveratrol-linkage networks by Cytoscape

**Figure 1:**
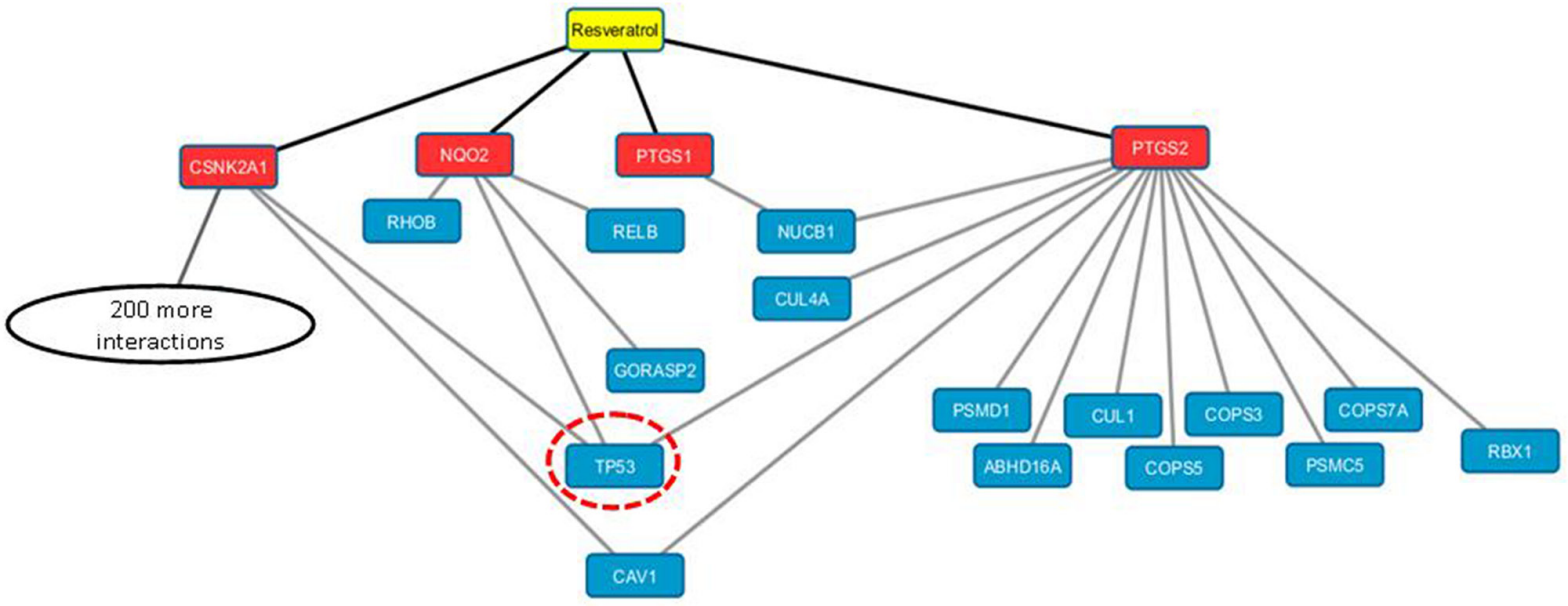
Drug-target interactome of resveratrol: DTome based resveratrol drug-target protein interaction network Drug: resveratrol (color in yellow), primary direct protein targets (DPT): CSNK2A1, NQO2, PTGS1 and PTGS2 (color in red) and secondary DPT-interacting protein: color in blue. p53 (circle in dash red) is targeted by 3 out of 4 resveratrol direct targets.

In biological systems, chemicals (presented as metabolites, drugs, or chemopreventive agents), proteins, and genes interact with one another to result in a physiologic function at the cellular and organ levels. This higher-order, complex interaction among molecules can be visualized as a network which typically consists of nodes representing biological entities (e.g., gene, protein, disease) and edges representing the relationships among them (e.g., physical interaction, co-expression or co-modulation, shared pathway). We first queried DTome using resveratrol as input. This resulted in output DB02709 categorizing resveratrol as antioxidants, platelet aggregation inhibitors, enzyme inhibitors, anti-carcinogenic agents, anti-neoplastic agents, phytogenic and anti-mutagenic agents. Moreover, using the grouping status of resveratrol as an experimental drug, query of DTome indicated that it is a chemical investigated for the treatment of herpes labialis infections (cold sores) (Table 1). Table 2 presents the 4 primary direct protein targets (DPT) of resveratrol, respectively, PTGS1, CSNK2A1, NQO2 and PTGS2. Expanding our search and analysis using the “Get protein-protein interaction (PPI) option” in DTome, we identified a total of 219 target-protein interactions designated DPT-associated genes as being related to resveratrol and its 4 primary targets (Appendix 1). The data set obtained was then collectively integrated for construction of a relevant biological network using Cytoscape 2.8 [17]. This resulted in the creation of a resveratrol network and the visualization of resveratrol target-protein interactions. The 4 primary DPTs and their secondary DPT-associated proteins, CSNK2A1 (202 PPI), NQO2 (4 PPI), PTGS1 (1 PPI) and PTGS2 (12 PPI), are shown in Figure 1. It should be noted that except for PTGS1, the other three primary targets show a direct association with control of tumor suppressor gene p53 suggesting that a main target pathway under control or mediated by resveratrol is connected to p53.

**Table 1:** Characterization of resveratrol using DTome

DB_ID	Name	Group	Category	Indication
DB02709	Resveratrol	Experimental	Antioxidants	Being investigated for the treatment of Herpes labialis infections (cold sores)
			Platelet aggregation inhibitors	
			Enzyme inhibitors	
			Anticarcinogenic agents	
			Antineoplastic agents	
			Phytogenic	
			Antimutagenic agents	

**Table 2:** Identification of direct targets of resveratrol using DTome

	Searched_Drug (1/1)		Target (4)		
#	DB_ID	Name	Target_Symbol	UniProtKB_AC	Entrez_ID
1	DB02709	Resveratrol	PTGS1	P23219	5742
2	DB02709	Resveratrol	CSNK2A1	P68400	1457
3	DB02709	Resveratrol	NQO2	P16083	4835
4	DB02709	Resveratrol	PTGS2	P35354	5743

### Analysis of functional attributes connected to resveratrol-mediated changes in gene sets using WebGestalt

To assess functional features of resveratrol-mediated gene sets, we performed the KEGG pathway enrichment analysis embedded in WebGestalt. The top 10 KEGG pathways linked to resveratrol DPTs and their DPT-associated genes include pathways in cancer (31 genes), cell cycle (15 genes), MAPK signaling (19 genes), Wnt signaling (15 genes), Neurotrophin signaling (12 genes), adherens junction (10 genes), prostate cancer (10 genes), p53 signaling (8 genes), epithelial cell signaling in *Helicobacter pylori* infection (8 genes) and melanogenesis (9 genes) (Table 3). All enriched pathways identified using this approach represent biological areas that show a statistically significant association with resveratrol gene sets thus warranting further investigation. Broad grouping of the functional analysis suggests that resveratrol associated genes are mainly linked to cancer-related and signaling cascade pathways with potential mechanistic underpinnings, including (i) control of cancer cell proliferation and survival via MAPK/p53 mediated cell cycle control, (ii) control of neural or cancer stem cell development via neurotrophin signaling, and (iii) regulation of gene transcription by Wnt/b-catenin signaling. Given that 3 of 4 primary targets of resveratrol have demonstrated a direct association with the p53 axis (Figure 1), emphasis was directed to the p53 signaling pathway. Further analyses revealed that 8 genes in the p53 pathway showed a connection to resveratrol-associated genes, PTEN, TP53, CHEK1, CDKN1A, BID, CDK1, SFN and IGFBP3 (EntrezGene ID: 5728, 7157, 1111, 1026, 637, 983, 2810 and 3486) (Table 3). Thus, based on the results of functional analysis combined with resveratrol target search, it may be suggested that a functional association exists between p53 and resveratrol thereby linking DPTs of resveratrol with p53 control. In addition, the enriched KEGG pathways derived from resveratrol gene sets also discovered 10 genes, CREBBP, HSP90B1, CTNNB1, PTEN, NFKBIA, TP53, HSP90AA1, CDKN1A, RELA and TCF7L2 (EntrezGene ID: 1387, 7184, 1499, 5728, 4792, 7157, 3320, 1026, 5970 and 6934), all associated with prostate cancer (Table 3). It is noteworthy that anti-carcinogenic properties of resveratrol have been observed in other solid tumors including the highly fatal pancreatic cancer [18, 19]. Evidence has also been garnered recently that resveratrol demonstrates efficacy in the treatment of inflammation-induced pancreatitis [20, 21].

**Table 3:** List of enriched resveratrol associated gene sets identified using KEGG PATHWAYS ANALYSIS

Pathway Name	#Gen	Entrez Gene (corresponding gene set)	Statistics
Pathways in cancer	31	7184 4149 2247 9978 7157 1857 324 1026 2353 637 6934 2246 1387 3725 5743 1499 999 5728 3091 4609 35764792 5371 3065 3320 1855 6688 5970 405 1856 3066	C=326;O=31;E=1.65;R=18.81; rawP=4.58e-30;adjP=4.53e-28
Cell cycle	15	1387 7465 9978 4609 7157 10971 8454 3065 1111 1026 994 983 7529 2810 3066	C=124;O=15;E=0.63;R=23.93; rawP=1.08e-16;adjP=5.35e-15
MAPK signaling pathway	19	5058 4149 2247 7157 2353 3925 1432 3725 2246 4609 408 5971 6722 994 409 5970 1386 1649 4208	C=268;O=19;E=1.35;R=14.03; rawP=2.05e-16;adjP=6.77e-15
Wnt signaling pathway	15	3725 1387 1499 9978 4609 7157 8454 1457 1857 1460 324 1855 1856 1459 6934	C=150;O=15;E=0.76;R=19.78; rawP=1.97e-15;adjP=4.88e-14
Neurotrophin signaling pathway	12	3667 3725 805 4792 801 7157 5664 10971 808 5970 1432 7529	C=127;O=12;E=0.64;R=18.69; rawP=2.65e-12;adjP=5.25e-11
Adherens junction	10	1387 5770 7454 999 1499 1457 1460 6714 1459 6934	C=73;O=10;E=0.37;R=27.10; rawP=4.17e-12;adjP=6.88e-11
Prostate cancer	10	1387 7184 1499 5728 4792 7157 3320 1026 5970 6934	C=89;O=10;E=0.45;R=22.23; rawP=3.18e-11;adjP=4.50e-10
p53 signaling pathway	8	5728 7157 1111 1026 637 983 2810 3486	C=68;O=8;E=0.34;R=23.27; rawP=2.13e-09;adjP=2.34e-08
Epithelial cell signaling in Helicobacter pylori infection	8	5058 3725 50848 5970 1432 6714 4792 3576	C=68;O=8;E=0.34;R=23.27; rawP=2.13e-09;adjP=2.34e-08
Melanogenesis	9	1387 805 1499 801 808 1857 1855 6934 1856	C=101;O=9;E=0.51;R=17.63; rawP=2.55e-09;adjP=2.52e-08

The row lists the following statistics: C: the number of reference genes in the category; O: the number of genes in the gene set and also in the category; E: the expected number in the category; R: ratio of enrichment; rawP: *p* value from hypergeometric test; adjP: *p* value adjusted by the multiple test adjustment

### Mining genetic alterations connected with resveratrol-associated genes, PTEN, TP53 and CDKN1A, in prostate cancer by cBio portal

**Figure 2:**
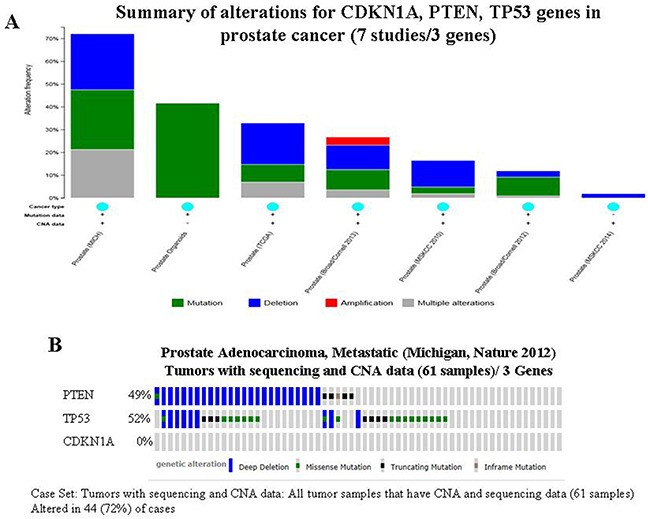
Mining genetic alterations connected with resveratrol-associated genes, PTEN, TP53 and CDKN1A, in prostate cancer studies embedded in cBio cancer genomics portal **A.** Overview of changes on PTEN, TP53 and CDKN1A genes in genomics data sets available in 7 different prostate cancer studies. **B.** OncoPrint: A visual summary of alteration across a set of prostate samples (data taken from the Michigan studies, Nature 2012) [24] based on a query of the three genes PTEN, TP53 and CDKN1A). Distinct genomic alterations including mutations and CNAs (copy number alterations, exemplified by gene amplifications and homozygous deletions) are summarized and color coded presented by % changes in particular affected genes in individual tumor samples. Each row represents a gene, and each column represents a tumor sample. Red bars designate gene amplifications, blue bars represent homozygous deletions, and green squares indicate nonsynonymous mutations.

Although DTome proffers resveratrol as the choice of treatment for infections (Table 1); the functional enrichment analysis uncovers the link between resveratrol associated genes and cancer-related pathways (Table 3). To further explore the validity of this link, cBio portal, a web-based integrated data mining system was used to explore the genetic alteration of genes associated with resveratrol in prostate cancer. Since the p53 signaling pathway is a main target of resveratrol, and because 3 overlapping genes (PTEN, TP53 and CDKN1A) were also found to be associated with prostate cancer by KEGG analysis embedded in WebGestalt, we utilized the 3 indicated overlapping genes identified in both p53 signaling and KEGG pathways to cross check their cancer genomic alterations and clinical profiles in prostate cancer (Table 3). A query of 3 selected overlapping genes was performed. Among 7 prostate cancer studies analyzed [22–27], alterations ranging from 1.9% to 72.1% were found for the gene sets/pathways submitted for analysis (Figure 2A). A summary of the multiple gene alterations observed across each set of tumor samples from the Michigan study [24] showing the most pronounced genomic changes is presented using OncoPrint. The results show that 44 cases (72%) have an alteration in at least one of the three genes queried; the frequency of alteration in each of the selected genes is shown in Figure 2B. No gene alteration occurred in CDKN1A (Figure 2B). For PTEN, most alterations are classified as deep deletions with a few cases of mutations (Figure 2B). Gene changes associated with TP53 include deep deletions and missense/truncating mutations (Figure 2B). The alterations in these two genes tend towards co-occurrence across samples, however, Mutual Exclusivity analysis showed no statistical significance (p = 0.183) (data not shown).

**Figure 3:**
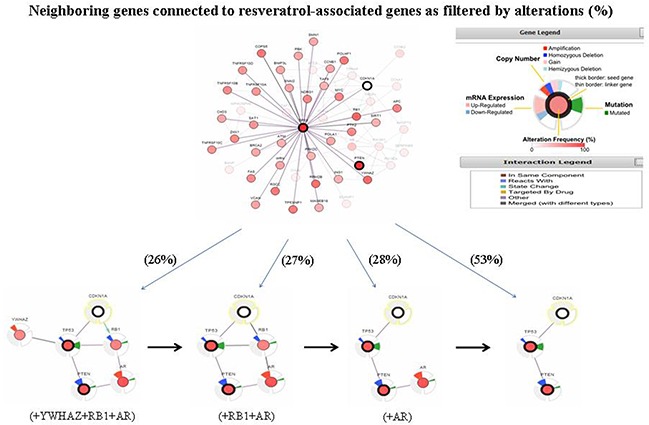
A visual display of the gene network connected to PTEN/TP53/CDKN1A in prostate adenocarcinoma (based on the Michigan study, Nature 2012) [24] The data mined from the cBio cancer genomics portal. PTEN, TP53, CDKN1A, and resveratrol associated genes, are used as seed genes (indicated with thick black border), to automatically harvest all other genes identified as altered in prostate adenocarcinoma (taken from the Michigan study, Nature 2012). Multidimensional genomic details are shown for seed genes PTEN, TP53 and CDKN1A. Darker red indicates increased frequency of alteration (defined by mutation, copy number amplification, or homozygous deletion) in prostate cancer. Shown in the figure is the full and pruned network containing all or partial neighbors of all query genes generated; the filters used included highest genomic alteration frequency within the selected cancer study in addition to the query genes.

cBio portal also provides interactive analysis and construct networks to view genes that are altered in cancer. Here, we first show the network to contain all neighbors of 3 query genes, PTEN, TP53 and CDKN1A (Figure 3). Next, to reduce the complexity of network analysis, the genomic alteration frequency within the selected cancer study was applied as a filter such that only the neighbors with the highest alteration frequency in addition to our query genes are shown. This refined analysis generated a different complexity of altered gene set networks. As a start, the 3 overlapping genes (PTEN, TP53 and CDKN1A) were identified when neighbors' ≥53% alteration was applied as the filter. Comparatively, 4 genes including androgen receptor (AR) were evident using a filter of 28% alteration. A 5 gene cluster with AR and retinoblastoma (RB1) was observed with a filter incorporating a 27% alteration, and 6 genes with the addition of AR, RB1 and YWHAZ (tyrosine 3-monooxygenase/tryptophan 5-monooxygenase activation protein, zeta) were revealed when the filter was reduced to 26% alteration (Figure 3). The full and pruned networks generated show the potential of complexity as well as the variability of difference in interactions between resveratrol associated genes most relevant to the genes altered in prostate cancer tumor samples from the Michigan study [24].

## DISCUSSION

Since the landmark finding of the chemoprotective activity of resveratrol reported by Jang et al. [1]; more than 7600 publications on resveratrol have appeared on PubMed to date. A wide range of biological and cellular activities and multiple targets have been independently identified for resveratrol illustrating the fascinating nature of this compound with a plethora of associated diseases. Still, how resveratrol promotes its wide range of beneficial effects remains incomplete. As such, new analytical methods or platforms are needed capable of bridging resveratrol to its target proteins for linkage to the observed biological effects. In this study, we introduce FAN analysis to elucidate the molecular mechanisms of resveratrol and its association with clinical outcomes in cancer by using a set of web-based tools. FAN analysis explores the molecular action of resveratrol by querying multidimensional cancer genomics data embedded in two open platforms, DTome [16] and cBio portal [28]. This workflow combines two simple steps: (i) identify primary DPTs of resveratrol and functionally integrate with DPT-associated proteins using DTome [16] and (ii) explore and verify whether genetic alterations exist for the identified resveratrol-associated genes/proteins across samples in large-scale cancer genomics projects by cBio portal [28]. The FAN analysis enables researchers to interactively explore effective targets of resveratrol for connectivity to information about the biological pathways for all the genes linked to resveratrol identified DPTs and DPT-associated genes and ultimately to clinical outcomes — to our knowledge, this is a hitherto unexplored feature that translates data set from bench to the bedside, with significant certainty of biological plausibility. Moreover, FAN analysis also can generate research leads not merely driven by hypothesis but transcends to ‘rationalized hypotheses' where postulates designed to deduce biological pathways, in terms of gene regulatory networks or protein-protein interaction networks, are formulated by mining a single or multiple web-based integrated data systems. This approach differs significantly from use of experimental techniques for determining a few interactions at a time, and takes into account findings from perturbation studies showing that the suppression of one gene can cause the activity of numerous other genes to go up or down as implicated in the existence of a cellular network. By integrating and utilizing the existing available data sources, redundant experiments from the same or different laboratories can be reduced and research direction will be better guided.

In this paper, we demonstrate the feasibility of FAN analysis for connectivity between resveratrol and cancer using DTome (for searching the resveratrol target genes) combined with cBio portal (for mining alterations of resveratrol target genes in cancer). Analysis of resveratrol using these two Web-based resources identified 4 primary DPTs (PTGS1, CSNK2A1, NQO2 and PTGS2) (Table 2), 219 secondary DPT-associated genes/proteins (Appendix 1) and 10 enriched KEGG pathways linked to resveratrol associated genes (Table 3). Based on the known functional characteristics of 219 DPT-associated genes and our current knowledge of KEGG pathways, 31 genes may be considered as validated secondary targets of resveratrol with mechanistic connectivity to cancer (Table 3). We surmise that this diverse range of effects is initiated by the interplay between resveratrol and its primary DPTs positioned at the apex of many cellular events. Since 3 of 4 primary targets of resveratrol show direct association with p53 it is reasonable to suggest that effects of resveratrol are primarily mediated by the p53 axis (Figure 1). As support, Gene Set Enrichment analysis independently identifies the p53 signaling pathway as significantly altered by resveratrol (Table 3). The association between p53 and the beneficial effects exerted by resveratrol in cancer were further explored and evaluated by the genetic alterations in 3 overlapping genes (PTEN, TP53 and CDKN1A) revealed by resveratrol associated p53 signaling and prostate cancer pathways (Table 3). Both PTEN and p53 are tumor suppressor genes considered to be among the most commonly inactivated or mutated in human cancers. In the case of prostate cancer, most genetic alterations in PTEN and TP53 are deletions or mutations (Figure 2B), which result in a reduction of their expression in concordance with the acceleration of carcinogenesis [29, 30]. Studies published by us and others suggest that the anti-carcinogenic activities of resveratrol are coordinated with the activation of p53, concomitant with cell cycle control and induction of apoptosis [31–34]. Thus, results of FAN analysis are in good agreement with the activation of p53 by resveratrol as a major driver for the observed beneficial chemopreventive effects on prostate cancer. It is worth noting that 70% of men at the time of diagnosis with prostate cancer are estimated to have lost a copy of the PTEN gene [35]. Since p53 plays a crucial role in suppression of PTEN-deficient tumorigenesis via control of PTEN transcription [35], we propose that activation of p53 by resveratrol is likely to have chemopreventive efficacy in patients diagnosed with PTEN-deficient prostate cancer.

Another aspect of FAN analysis relevant to its utility and implications for the role resveratrol plays in prostate cancer relates to the three primary targets of resveratrol: CSNK2A1, NQO2 and PTGS2 (Table 2). Although they all show association with control of p53, it is not known whether they act as sensors, mediators, or capacitors of resveratrol. We suggest that DTome-flagged NQO2 is especially worth noting since it was independently purified and characterized by us as a high affinity resveratrol target protein (K_dis_=35 nM) using affinity chromatography and X-ray analysis [6]. A hypothesis currently being considered is that resveratrol acts through NQO2 to control prostate cancer cell proliferation and survival via regulated interaction with the p53 signaling axis. Ongoing studies in our laboratory are directed at charting the molecular pipeline emanating from resveratrol to NQO2 (its preferred target), followed by the p53 signaling network, and ultimately, the pipeline-defined cellular fate and phenotype. Demonstration of such a molecular pipeline initiated by resveratrol impinging on the p53 axis and control of prostate carcinogenesis should illuminate new biological insights on the control of cancer harboring dysfunctional p53.

In addition to prostate cancer, numerous studies have reported on the anti-tumorigenic and chemopreventive activities of resveratrol in other solid tumors including skin, breast, lung, colon, liver and pancreatic cancers [36]. Here, we presented FAN analysis and its utility to demonstrate the molecular network bridging the connectivity between resveratrol and its primary targets as well as resveratrol-associated genes; the functional link existing between the revealed pathways/network is likely to be biologically associated with the observed effects of resveratrol in disease of choice for the analysis, e.g., prostate cancer. Whether the connectivity map shown to exist between resveratrol and prostate cancer in this work can be extended to other solid tumors remains to be investigated. In principle, resveratrol-linkage network revealed by query of DTome could apply to a disease known to be efficaciously affected by resveratrol, provided that the inferred resveratrol-disease associations can be confirmed by disease association analysis in WebGestalt[16] (Appendix 2) as well as by cross-validation using disease-specific genetic gene expression profile studies [37]. Moreover, the FAN analysis could be further expanded to connect resveratrol-associated gene sets with cancer-linked, genetic alterations by mining cancer-specific databases like cBio Portal [21] and/or Oncomine [38, 39]. In addition, it is possible to gain insights on the role of resveratrol in the prevention of treatment of diseases by exploring alterations connected with resveratrol-associated genes by probing disease-specific gene expression signatures using data embedded in the Gene Expression Atlas of ArrayExpress or OMIN (Online Mendelian Inheritance in Man) — a comprehensive disease phenotype database of human genes and gene disorders [37, 40, 41].

Query of publicly available computational and database tools using the automated FAN analysis approach may significantly advance (i) understanding of malignant disease mechanisms, (ii) facilitate early disease diagnosis and improve the accuracy of disease prognosis, and (iii) unravel critical roles of resveratrol in human cancers. This approach is superior to the manual integration of information on resveratrol and its target genes which can be challenging, labor-intensive, and error-prone, possibly because an extremely large amount of heterogeneous data sources must be considered, tested and assayed. Comparatively, FAN analysis is completely machine-readable integrated from heterogeneous sources: DTome, KEGG pathway and cBio portal. FAN analysis can be used to obtain unified resveratrol-relevant knowledge and bring insights into the regulation and control of cancer disease processes. By providing a deeper understanding of resveratrol's functions, FAN analysis will also assist resveratrol bio-curation and new biological experiment design and could significantly accelerate cancer biology research. Although generally configured for providing primary genomic results rather than clinical associations, FAN analysis is extensible and can be readily generalized to other biomedical areas. The candidate genes revealed can facilitate the interpretation of genomic results for non-computational biomedical researchers and other users.

Lastly, one can surmise that FAN analysis may find application in the repurposing of drugs. Traditional drug development faces unique challenges mainly due to high cost and long duration for approval of use in clinical settings [42]. Accordingly, repositioning of existing drugs has taken on increasing appeal and importance as an alternative approach. Extensive pharmacokinetic and toxicological data also are available for marketed drugs, thus providing information which may be readily used to guide and position research, and for comparison of data generated in studies among laboratories.

Some difficulties remain in applying computational learning techniques to problems of predicting chemical/drug response. These challenges are appropriately summed up in one phrase: the intrinsic variability and difficulty of reproducibility of biological data. There is significant inherent nonrepeatability of experimental conditions and/or biological phenomena. Quality control on the extremely costly equipment used to make biological measurements can be a more insidious factor contributing to variability and lack of repeatability. To some degree, both factors can be in part counteracted by the FAN analysis.

In summary, as advances in research on resveratrol using traditional experimental techniques and approaches continue, more resveratrol targets will unquestionably be identified; nevertheless, the determination as to whether a specific biological effect on resveratrol can be definitively assigned to individual or simultaneous modulation of its newly or established identified targets can remain obscure. We believe that some of the uncertainty in connecting resveratrol to its targets and subsequent phenotype expression can be offset using FAN analysis, and that this approach can be used to direct future studies with reasonable experimental feasibility for testing and validating rationalized hypotheses regarding the effects of resveratrol in cancer. For example, a functional network can be developed based on the core elements of FAN analysis to further evaluate the impact of resveratrol as gauged by the changes in altered gene sets shown for a specific cancer type or cancer in general by expanding the complicity of cBio portal altered gene network (Figure 3). In addition, drug targets identified and experimentally verified through FAN analysis can be used for future drug development. Overall, FAN analysis provides a simple yet flexible interface to test hypotheses regarding genetic alterations in cancer by using existing drug information as a guiding BioGPS to aid researchers in translating basic research into clinical applications.

## MATERIALS AND METHODS

### Drug-target search

DTome is a web-based bioinformatic tool able to query the information embedded in three open-source databases, respectively, DrugBank, PharmGSK, and PINA; the data compiled are analyzed, grouped, and used to construct a drug-target interactome [16]. Interaction networks generated from DTome are integrated into four types of relationships: drug-drug interactions, drug-target interactions, drug-gene associations, and target-gene-protein interactions. In this study, DTome was used to search the interaction between resveratrol and its targets, to generate a resveratrol-target network. The data were then used to construct a visualization chart, followed by further analysis and the proposal of validation experiments.

### Network generation/visualization and gene set enrichment analysis

Through use of the DTome search function, drug-protein and second level protein-protein interactions data were first generated for resveratrol. The data were integrated into a resveratrol-mediated network and analyzed using Cytoscape (version 2.8.0) [17]. The biological information and attribution embedded in the generated gene set were analyzed using a web-based integrated data mining system, WebGestalt [43, 44]. Biochemical pathways and functions linked to the resveratrol gene set were specifically queried and navigated by the KEGG pathway enrichment analysis tool in WebGestalt [43]. The gene set enrichment analysis searches for combinations of features that show a significant difference in means between two classes that is not an artifact or data noise [45]. Significance in the identified gene sets is confirmed by replacing chosen significant features (i.e., gene sets) with other genes chosen at random, followed by testing and re-analyzing to see whether the significant difference still persists. The validated significant gene sets were then organized based on the KEGG biochemical pathways in a KEGG Table. Top 10 pathways with an adjusted P-value less than 0.01 were selected.

### Exploring cancer genomics data linked to resveratrol by cBio Cancer Genomics Portal

The cBio Cancer Genomics Portal (http://cbioportal.org) is an open platform for exploring multidimensional cancer genomics data by encapsulating molecular profiling data obtained from cancer tissues and cell lines into readily understandable genetic, epigenetic, gene expression, and proteomic events [28]. Complex cancer genomics profiles can be easily accessed using the query interface of the portal enabling researchers to explore and compare genetic alterations across samples. The underlying data thus obtained can be linked to clinical outcomes to facilitate novel discovery in biological systems.

In this study, the cBio Portal was used to explore the connectivity of resveratrol associated genes across all prostate cancer studies available in the databases. Through use of the portal search function, resveratrol associated genes in all samples of prostate cancer studies are classified as altered or not altered. The genomics datasets are then presented using OncoPrint as heatmaps — a visually appealing display of alterations in gene arrays across tumor samples [46]. Another feature of the portal is that it can generate multiple visualization platforms by grouping prostate cancer data alterations using input from resveratrol gene sets [46–50].

## SUPPLEMENTARY FIGURES AND TABLES




