# Supplementary material for: Functional/activity network (FAN) analysis of gene-phenotype connectivity liaised by grape polyphenol resveratrol
Source: Oncotarget. 2016 May 24;7(25):38670–80. doi: 10.18632/oncotarget.9578 (PMC5122419; doi:10.18632/oncotarget.9578)
Supplement: Supplementary file 1 [file oncotarget-07-38670-s001.pdf]

SUPPLEMENTARY APPENDIX

Supplementary Appendix 1: Output of search and analysis by DTome on 4 primary direct protein targets (DPT) of resveratrol and DPT-associated protein-protein interaction (PPI) related to resveratrol

See Supplementary File 1

Supplementary Appendix 2: WebGestalt Enrichment analysis for resveratrol and the list of resveratrol target genes in the top 10 diseases identified in the analysis

Disease	#Gene	EntrezGene	Statistics
Cancer or viral infections	50	8091 3958 1958 3217 7157 3978 324 1111 1026 9656 637 9049 6714 7153 3725 6696 1499 999 5728 4609 5300 835 4613 3320 52415970 6688 4602 3486 6667 5058 328 672 857 2353 983 7515 931 4255 5743 3091 5371 3065 6198 3159 7518 2810 4311 3066 1649	C=951;O=50;E=4.81;R=10.40; rawP=7.79e-36;adjP=5.30e-33
Drug interaction with drug	32	7157 25942 1432 637 6714 3725 1387 7153 1499 7448 5300 653361 3320 2288 5970 11140 7150 6667 2247 801 55690 4691 857 23536622 6804 5524 408 3065 409 405 3066	C=349;O=32;E=1.76;R=18.14; rawP=1.65e-30;adjP=5.62e-28
Stress	32	10808 7184 1958 3297 7157 845 1111 1026 3163 9656 1432 6125 3725 5300 835 994 3320 1386 5970 5058 328 4835 4691 2353 3886622 3091 1978 4792 3576 5371 1649	C=464;O=32;E=2.35;R=13.64; rawP=1.34e-26;adjP=3.04e-24
Neoplasms	37	8091 3958 1958 3217 7157 324 1026 3925 9049 6714 7153 6696 1499 999 5728 4609 5300 4613 5241 6688 4602 3486 328 672 857 9317515 4255 5743 3091 5371 6198 3159 7518 4311 2810 1649	C=854;O=37;E=4.32;R=8.57; rawP=1.42e-23;adjP=2.42e-21
Breast Neoplasms	27	3958 3217 7157 1111 1026 8996 6714 7153 6696 84152 1499 999 5728 4609 5300 5241 4602 3486 5058 857 672 7515 5743 3091 6198 2810 3066	C=377;O=27;E=1.91;R=14.17; rawP=5.39e-23;adjP=7.34e-21

(*Continued*)

Disease	#Gene	EntrezGene	Statistics
Li-Fraumeni syndrome	20	7157 1111 324 1026 25942 1387 10923 1499 5728 4609 5970 6667 672 7515 4255 1107 5371 3065 2810 3428	C=171;O=20;E=0.86;R=23.14; rawP=1.48e-21;adjP=1.68e-19
Breast Diseases	25	3958 3217 7157 1111 1026 6714 7153 6696 84152 1499 999 5728 4609 5300 5241 3486 5058 857 672 7515 5743 3091 6198 2810 3066	C=350;O=25;E=1.77;R=14.13; rawP=2.47e-21;adjP=2.40e-19
HIV	33	9150 5684 8451 6872 1457 808 1460 1432 3725 1387 805 7465 3603 5707 5511 5971 1212 5970 1459 7150 6667 5058 801 55690 23535685 983 5788 4792 3576 5524 3159 5705	C=755;O=33;E=3.82;R=8.65; rawP=3.36e-21;adjP=2.86e-19
Head and Neck Neoplasms	20	8091 7184 3958 7157 324 1026 6696 999 1499 5728 5300 4609 4602 2247 7515 4255 5743 3091 10987 2810	C=262;O=20;E=1.32;R=15.10; rawP=7.61e-18;adjP=5.76e-16
Cell Transformation, Neoplastic	19	8091 4149 3958 3217 7157 1026 6714 7258 999 1499 5728 5300 4609 4613 4602 5058 2353 5371 3159	C=233;O=19;E=1.18;R=16.13; rawP=1.51e-17;adjP=1.03e-15

The row lists the following statistics: C: the number of reference genes in the category; O: the number of genes in the gene set and also in the category; E: the expected number in the category; R: ratio of enrichment; rawP: *p* value from hypergeometric test; adjP: *p* value adjusted by the multiple test adjustment
