# Supplementary material for: Functional/activity network (FAN) analysis of gene-phenotype connectivity liaised by grape polyphenol resveratrol
Source: Oncotarget. 2016 May 24;7(25):38670–80. doi: 10.18632/oncotarget.9578 (PMC5122419; doi:10.18632/oncotarget.9578)
Supplement: Supplementary file 2 [file oncotarget-07-38670-s002.doc]

**SupplementaryAppendix 1: Output of search and analysis by DTome on 4 primary direct protein targets (DPT) of resveratrol and DPT-associated protein-protein interaction (PPI) related to resveratrol**

|  | **Searched_Drug ( 1/1 )** | | **PPI (219)** | | | |
| --- | --- | --- | --- | --- | --- | --- |
| **#** | **DB_ID** | **Name** | **Target_ID** | **Target_Symbol** | **PPI_ID** | **PPI_Symbol** |
| 1 | DB02709 | Resveratrol | 1457 | CSNK2A1 | 100289087 | TSPY10 |
| 2 | DB02709 | Resveratrol | 1457 | CSNK2A1 | 1026 | CDKN1A |
| 3 | DB02709 | Resveratrol | 1457 | CSNK2A1 | 10287 | RGS19 |
| 4 | DB02709 | Resveratrol | 1457 | CSNK2A1 | 10428 | CFDP1 |
| 5 | DB02709 | Resveratrol | 1457 | CSNK2A1 | 10445 | MCRS1 |
| 6 | DB02709 | Resveratrol | 1457 | CSNK2A1 | 10661 | KLF1 |
| 7 | DB02709 | Resveratrol | 1457 | CSNK2A1 | 10749 | KIF1C |
| 8 | DB02709 | Resveratrol | 1457 | CSNK2A1 | 10808 | HSPH1 |
| 9 | DB02709 | Resveratrol | 1457 | CSNK2A1 | 10921 | RNPS1 |
| 10 | DB02709 | Resveratrol | 1457 | CSNK2A1 | 10923 | SUB1 |
| 11 | DB02709 | Resveratrol | 1457 | CSNK2A1 | 10971 | YWHAQ |
| 12 | DB02709 | Resveratrol | 1457 | CSNK2A1 | 11036 | GTF2A1L |
| 13 | DB02709 | Resveratrol | 1457 | CSNK2A1 | 1107 | CHD3 |
| 14 | DB02709 | Resveratrol | 1457 | CSNK2A1 | 1111 | CHEK1 |
| 15 | DB02709 | Resveratrol | 1457 | CSNK2A1 | 11124 | FAF1 |
| 16 | DB02709 | Resveratrol | 1457 | CSNK2A1 | 11140 | CDC37 |
| 17 | DB02709 | Resveratrol | 1457 | CSNK2A1 | 11244 | ZHX1 |
| 18 | DB02709 | Resveratrol | 1457 | CSNK2A1 | 1212 | CLTB |
| 19 | DB02709 | Resveratrol | 1457 | CSNK2A1 | 1386 | ATF2 |
| 20 | DB02709 | Resveratrol | 1457 | CSNK2A1 | 1387 | CREBBP |
| 21 | DB02709 | Resveratrol | 1457 | CSNK2A1 | 1390 | CREM |
| 22 | DB02709 | Resveratrol | 1457 | CSNK2A1 | 140890 | SREK1 |
| 23 | DB02709 | Resveratrol | 1457 | CSNK2A1 | 1432 | MAPK14 |
| 24 | DB02709 | Resveratrol | 1457 | CSNK2A1 | 1459 | CSNK2A2 |
| 25 | DB02709 | Resveratrol | 1457 | CSNK2A1 | 1460 | CSNK2B |
| 26 | DB02709 | Resveratrol | 1457 | CSNK2A1 | 1482 | NKX2-5 |
| 27 | DB02709 | Resveratrol | 1457 | CSNK2A1 | 1499 | CTNNB1 |
| 28 | DB02709 | Resveratrol | 1457 | CSNK2A1 | 1649 | DDIT3 |
| 29 | DB02709 | Resveratrol | 1457 | CSNK2A1 | 1855 | DVL1 |
| 30 | DB02709 | Resveratrol | 1457 | CSNK2A1 | 1856 | DVL2 |
| 31 | DB02709 | Resveratrol | 1457 | CSNK2A1 | 1857 | DVL3 |
| 32 | DB02709 | Resveratrol | 1457 | CSNK2A1 | 19 | ABCA1 |
| 33 | DB02709 | Resveratrol | 1457 | CSNK2A1 | 1933 | EEF1B2 |
| 34 | DB02709 | Resveratrol | 1457 | CSNK2A1 | 1936 | EEF1D |
| 35 | DB02709 | Resveratrol | 1457 | CSNK2A1 | 1958 | EGR1 |
| 36 | DB02709 | Resveratrol | 1457 | CSNK2A1 | 1978 | EIF4EBP1 |
| 37 | DB02709 | Resveratrol | 1457 | CSNK2A1 | 1983 | EIF5 |
| 38 | DB02709 | Resveratrol | 1457 | CSNK2A1 | 2079 | ERH |
| 39 | DB02709 | Resveratrol | 1457 | CSNK2A1 | 2153 | F5 |
| 40 | DB02709 | Resveratrol | 1457 | CSNK2A1 | 2246 | FGF1 |
| 41 | DB02709 | Resveratrol | 1457 | CSNK2A1 | 2247 | FGF2 |
| 42 | DB02709 | Resveratrol | 1457 | CSNK2A1 | 2287 | FKBP3 |
| 43 | DB02709 | Resveratrol | 1457 | CSNK2A1 | 2288 | FKBP4 |
| 44 | DB02709 | Resveratrol | 1457 | CSNK2A1 | 2312 | FLG |
| 45 | DB02709 | Resveratrol | 1457 | CSNK2A1 | 23316 | CUX2 |
| 46 | DB02709 | Resveratrol | 1457 | CSNK2A1 | 2353 | FOS |
| 47 | DB02709 | Resveratrol | 1457 | CSNK2A1 | 2569 | GABRR1 |
| 48 | DB02709 | Resveratrol | 1457 | CSNK2A1 | 2570 | GABRR2 |
| 49 | DB02709 | Resveratrol | 1457 | CSNK2A1 | 25942 | SIN3A |
| 50 | DB02709 | Resveratrol | 1457 | CSNK2A1 | 25959 | KANK2 |
| 51 | DB02709 | Resveratrol | 1457 | CSNK2A1 | 2596 | GAP43 |
| 52 | DB02709 | Resveratrol | 1457 | CSNK2A1 | 26353 | HSPB8 |
| 53 | DB02709 | Resveratrol | 1457 | CSNK2A1 | 2764 | GMFB |
| 54 | DB02709 | Resveratrol | 1457 | CSNK2A1 | 2810 | SFN |
| 55 | DB02709 | Resveratrol | 1457 | CSNK2A1 | 2821 | GPI |
| 56 | DB02709 | Resveratrol | 1457 | CSNK2A1 | 2957 | GTF2A1 |
| 57 | DB02709 | Resveratrol | 1457 | CSNK2A1 | 2972 | BRF1 |
| 58 | DB02709 | Resveratrol | 1457 | CSNK2A1 | 2997 | GYS1 |
| 59 | DB02709 | Resveratrol | 1457 | CSNK2A1 | 3059 | HCLS1 |
| 60 | DB02709 | Resveratrol | 1457 | CSNK2A1 | 3065 | HDAC1 |
| 61 | DB02709 | Resveratrol | 1457 | CSNK2A1 | 3066 | HDAC2 |
| 62 | DB02709 | Resveratrol | 1457 | CSNK2A1 | 3091 | HIF1A |
| 63 | DB02709 | Resveratrol | 1457 | CSNK2A1 | 31 | ACACA |
| 64 | DB02709 | Resveratrol | 1457 | CSNK2A1 | 3159 | HMGA1 |
| 65 | DB02709 | Resveratrol | 1457 | CSNK2A1 | 3163 | HMOX2 |
| 66 | DB02709 | Resveratrol | 1457 | CSNK2A1 | 3181 | HNRNPA2B1 |
| 67 | DB02709 | Resveratrol | 1457 | CSNK2A1 | 3183 | HNRNPC |
| 68 | DB02709 | Resveratrol | 1457 | CSNK2A1 | 3217 | HOXB7 |
| 69 | DB02709 | Resveratrol | 1457 | CSNK2A1 | 324 | APC |
| 70 | DB02709 | Resveratrol | 1457 | CSNK2A1 | 328 | APEX1 |
| 71 | DB02709 | Resveratrol | 1457 | CSNK2A1 | 3297 | HSF1 |
| 72 | DB02709 | Resveratrol | 1457 | CSNK2A1 | 3320 | HSP90AA1 |
| 73 | DB02709 | Resveratrol | 1457 | CSNK2A1 | 3428 | IFI16 |
| 74 | DB02709 | Resveratrol | 1457 | CSNK2A1 | 3486 | IGFBP3 |
| 75 | DB02709 | Resveratrol | 1457 | CSNK2A1 | 353515 | XKRY2 |
| 76 | DB02709 | Resveratrol | 1457 | CSNK2A1 | 3576 | IL8 |
| 77 | DB02709 | Resveratrol | 1457 | CSNK2A1 | 3603 | IL16 |
| 78 | DB02709 | Resveratrol | 1457 | CSNK2A1 | 361 | AQP4 |
| 79 | DB02709 | Resveratrol | 1457 | CSNK2A1 | 3660 | IRF2 |
| 80 | DB02709 | Resveratrol | 1457 | CSNK2A1 | 3667 | IRS1 |
| 81 | DB02709 | Resveratrol | 1457 | CSNK2A1 | 3725 | JUN |
| 82 | DB02709 | Resveratrol | 1457 | CSNK2A1 | 3800 | KIF5C |
| 83 | DB02709 | Resveratrol | 1457 | CSNK2A1 | 3897 | L1CAM |
| 84 | DB02709 | Resveratrol | 1457 | CSNK2A1 | 39 | ACAT2 |
| 85 | DB02709 | Resveratrol | 1457 | CSNK2A1 | 3925 | STMN1 |
| 86 | DB02709 | Resveratrol | 1457 | CSNK2A1 | 3958 | LGALS3 |
| 87 | DB02709 | Resveratrol | 1457 | CSNK2A1 | 3978 | LIG1 |
| 88 | DB02709 | Resveratrol | 1457 | CSNK2A1 | 405 | ARNT |
| 89 | DB02709 | Resveratrol | 1457 | CSNK2A1 | 407 | ARR3 |
| 90 | DB02709 | Resveratrol | 1457 | CSNK2A1 | 408 | ARRB1 |
| 91 | DB02709 | Resveratrol | 1457 | CSNK2A1 | 409 | ARRB2 |
| 92 | DB02709 | Resveratrol | 1457 | CSNK2A1 | 4149 | MAX |
| 93 | DB02709 | Resveratrol | 1457 | CSNK2A1 | 4150 | MAZ |
| 94 | DB02709 | Resveratrol | 1457 | CSNK2A1 | 4208 | MEF2C |
| 95 | DB02709 | Resveratrol | 1457 | CSNK2A1 | 4255 | MGMT |
| 96 | DB02709 | Resveratrol | 1457 | CSNK2A1 | 4311 | MME |
| 97 | DB02709 | Resveratrol | 1457 | CSNK2A1 | 4602 | MYB |
| 98 | DB02709 | Resveratrol | 1457 | CSNK2A1 | 4609 | MYC |
| 99 | DB02709 | Resveratrol | 1457 | CSNK2A1 | 4613 | MYCN |
| 100 | DB02709 | Resveratrol | 1457 | CSNK2A1 | 4617 | MYF5 |
| 101 | DB02709 | Resveratrol | 1457 | CSNK2A1 | 466 | ATF1 |
| 102 | DB02709 | Resveratrol | 1457 | CSNK2A1 | 4676 | NAP1L4 |
| 103 | DB02709 | Resveratrol | 1457 | CSNK2A1 | 4691 | NCL |
| 104 | DB02709 | Resveratrol | 1457 | CSNK2A1 | 4792 | NFKBIA |
| 105 | DB02709 | Resveratrol | 1457 | CSNK2A1 | 5048 | PAFAH1B1 |
| 106 | DB02709 | Resveratrol | 1457 | CSNK2A1 | 5058 | PAK1 |
| 107 | DB02709 | Resveratrol | 1457 | CSNK2A1 | 50848 | F11R |
| 108 | DB02709 | Resveratrol | 1457 | CSNK2A1 | 51177 | PLEKHO1 |
| 109 | DB02709 | Resveratrol | 1457 | CSNK2A1 | 5176 | SERPINF1 |
| 110 | DB02709 | Resveratrol | 1457 | CSNK2A1 | 5241 | PGR |
| 111 | DB02709 | Resveratrol | 1457 | CSNK2A1 | 5300 | PIN1 |
| 112 | DB02709 | Resveratrol | 1457 | CSNK2A1 | 5303 | PIN4 |
| 113 | DB02709 | Resveratrol | 1457 | CSNK2A1 | 5371 | PML |
| 114 | DB02709 | Resveratrol | 1457 | CSNK2A1 | 5473 | PPBP |
| 115 | DB02709 | Resveratrol | 1457 | CSNK2A1 | 54926 | UBE2R2 |
| 116 | DB02709 | Resveratrol | 1457 | CSNK2A1 | 5504 | PPP1R2 |
| 117 | DB02709 | Resveratrol | 1457 | CSNK2A1 | 5511 | PPP1R8 |
| 118 | DB02709 | Resveratrol | 1457 | CSNK2A1 | 5524 | PPP2R4 |
| 119 | DB02709 | Resveratrol | 1457 | CSNK2A1 | 55502 | HES6 |
| 120 | DB02709 | Resveratrol | 1457 | CSNK2A1 | 55690 | PACS1 |
| 121 | DB02709 | Resveratrol | 1457 | CSNK2A1 | 55814 | BDP1 |
| 122 | DB02709 | Resveratrol | 1457 | CSNK2A1 | 5621 | PRNP |
| 123 | DB02709 | Resveratrol | 1457 | CSNK2A1 | 5664 | PSEN2 |
| 124 | DB02709 | Resveratrol | 1457 | CSNK2A1 | 5684 | PSMA3 |
| 125 | DB02709 | Resveratrol | 1457 | CSNK2A1 | 5685 | PSMA4 |
| 126 | DB02709 | Resveratrol | 1457 | CSNK2A1 | 5728 | PTEN |
| 127 | DB02709 | Resveratrol | 1457 | CSNK2A1 | 57599 | WDR48 |
| 128 | DB02709 | Resveratrol | 1457 | CSNK2A1 | 5770 | PTPN1 |
| 129 | DB02709 | Resveratrol | 1457 | CSNK2A1 | 5788 | PTPRC |
| 130 | DB02709 | Resveratrol | 1457 | CSNK2A1 | 5905 | RANGAP1 |
| 131 | DB02709 | Resveratrol | 1457 | CSNK2A1 | 5970 | RELA |
| 132 | DB02709 | Resveratrol | 1457 | CSNK2A1 | 6125 | RPL5 |
| 133 | DB02709 | Resveratrol | 1457 | CSNK2A1 | 6198 | RPS6KB1 |
| 134 | DB02709 | Resveratrol | 1457 | CSNK2A1 | 6236 | RRAD |
| 135 | DB02709 | Resveratrol | 1457 | CSNK2A1 | 6303 | SAT1 |
| 136 | DB02709 | Resveratrol | 1457 | CSNK2A1 | 637 | BID |
| 137 | DB02709 | Resveratrol | 1457 | CSNK2A1 | 6418 | SET |
| 138 | DB02709 | Resveratrol | 1457 | CSNK2A1 | 6473 | SHOX |
| 139 | DB02709 | Resveratrol | 1457 | CSNK2A1 | 653361 | NCF1 |
| 140 | DB02709 | Resveratrol | 1457 | CSNK2A1 | 6571 | SLC18A2 |
| 141 | DB02709 | Resveratrol | 1457 | CSNK2A1 | 6622 | SNCA |
| 142 | DB02709 | Resveratrol | 1457 | CSNK2A1 | 6667 | SP1 |
| 143 | DB02709 | Resveratrol | 1457 | CSNK2A1 | 6688 | SPI1 |
| 144 | DB02709 | Resveratrol | 1457 | CSNK2A1 | 6689 | SPIB |
| 145 | DB02709 | Resveratrol | 1457 | CSNK2A1 | 6696 | SPP1 |
| 146 | DB02709 | Resveratrol | 1457 | CSNK2A1 | 6711 | SPTBN1 |
| 147 | DB02709 | Resveratrol | 1457 | CSNK2A1 | 6714 | SRC |
| 148 | DB02709 | Resveratrol | 1457 | CSNK2A1 | 672 | BRCA1 |
| 149 | DB02709 | Resveratrol | 1457 | CSNK2A1 | 6722 | SRF |
| 150 | DB02709 | Resveratrol | 1457 | CSNK2A1 | 6749 | SSRP1 |
| 151 | DB02709 | Resveratrol | 1457 | CSNK2A1 | 6804 | STX1A |
| 152 | DB02709 | Resveratrol | 1457 | CSNK2A1 | 6872 | TAF1 |
| 153 | DB02709 | Resveratrol | 1457 | CSNK2A1 | 6934 | TCF7L2 |
| 154 | DB02709 | Resveratrol | 1457 | CSNK2A1 | 7054 | TH |
| 155 | DB02709 | Resveratrol | 1457 | CSNK2A1 | 715 | C1R |
| 156 | DB02709 | Resveratrol | 1457 | CSNK2A1 | 7150 | TOP1 |
| 157 | DB02709 | Resveratrol | 1457 | CSNK2A1 | 7153 | TOP2A |
| 158 | DB02709 | Resveratrol | 1457 | CSNK2A1 | 7157 | TP53 |
| 159 | DB02709 | Resveratrol | 1457 | CSNK2A1 | 7184 | HSP90B1 |
| 160 | DB02709 | Resveratrol | 1457 | CSNK2A1 | 7258 | TSPY1 |
| 161 | DB02709 | Resveratrol | 1457 | CSNK2A1 | 728137 | TSPY3 |
| 162 | DB02709 | Resveratrol | 1457 | CSNK2A1 | 7343 | UBTF |
| 163 | DB02709 | Resveratrol | 1457 | CSNK2A1 | 7421 | VDR |
| 164 | DB02709 | Resveratrol | 1457 | CSNK2A1 | 7448 | VTN |
| 165 | DB02709 | Resveratrol | 1457 | CSNK2A1 | 7454 | WAS |
| 166 | DB02709 | Resveratrol | 1457 | CSNK2A1 | 7465 | WEE1 |
| 167 | DB02709 | Resveratrol | 1457 | CSNK2A1 | 7504 | XK |
| 168 | DB02709 | Resveratrol | 1457 | CSNK2A1 | 7515 | XRCC1 |
| 169 | DB02709 | Resveratrol | 1457 | CSNK2A1 | 7518 | XRCC4 |
| 170 | DB02709 | Resveratrol | 1457 | CSNK2A1 | 7529 | YWHAB |
| 171 | DB02709 | Resveratrol | 1457 | CSNK2A1 | 79101 | TAF1D |
| 172 | DB02709 | Resveratrol | 1457 | CSNK2A1 | 801 | CALM1 |
| 173 | DB02709 | Resveratrol | 1457 | CSNK2A1 | 805 | CALM2 |
| 174 | DB02709 | Resveratrol | 1457 | CSNK2A1 | 808 | CALM3 |
| 175 | DB02709 | Resveratrol | 1457 | CSNK2A1 | 8091 | HMGA2 |
| 176 | DB02709 | Resveratrol | 1457 | CSNK2A1 | 835 | CASP2 |
| 177 | DB02709 | Resveratrol | 1457 | CSNK2A1 | 84152 | PPP1R1B |
| 178 | DB02709 | Resveratrol | 1457 | CSNK2A1 | 84232 | MAF1 |
| 179 | DB02709 | Resveratrol | 1457 | CSNK2A1 | 845 | CASQ2 |
| 180 | DB02709 | Resveratrol | 1457 | CSNK2A1 | 8473 | OGT |
| 181 | DB02709 | Resveratrol | 1457 | CSNK2A1 | 8479 | HIRIP3 |
| 182 | DB02709 | Resveratrol | 1457 | CSNK2A1 | 857 | CAV1 |
| 183 | DB02709 | Resveratrol | 1457 | CSNK2A1 | 8665 | EIF3F |
| 184 | DB02709 | Resveratrol | 1457 | CSNK2A1 | 8667 | EIF3H |
| 185 | DB02709 | Resveratrol | 1457 | CSNK2A1 | 8674 | VAMP4 |
| 186 | DB02709 | Resveratrol | 1457 | CSNK2A1 | 8841 | HDAC3 |
| 187 | DB02709 | Resveratrol | 1457 | CSNK2A1 | 8893 | EIF2B5 |
| 188 | DB02709 | Resveratrol | 1457 | CSNK2A1 | 8894 | EIF2S2 |
| 189 | DB02709 | Resveratrol | 1457 | CSNK2A1 | 8996 | NOL3 |
| 190 | DB02709 | Resveratrol | 1457 | CSNK2A1 | 9049 | AIP |
| 191 | DB02709 | Resveratrol | 1457 | CSNK2A1 | 9082 | XKRY |
| 192 | DB02709 | Resveratrol | 1457 | CSNK2A1 | 9150 | CTDP1 |
| 193 | DB02709 | Resveratrol | 1457 | CSNK2A1 | 9158 | FIBP |
| 194 | DB02709 | Resveratrol | 1457 | CSNK2A1 | 921 | CD5 |
| 195 | DB02709 | Resveratrol | 1457 | CSNK2A1 | 9221 | NOLC1 |
| 196 | DB02709 | Resveratrol | 1457 | CSNK2A1 | 931 | MS4A1 |
| 197 | DB02709 | Resveratrol | 1457 | CSNK2A1 | 9311 | ACCN3 |
| 198 | DB02709 | Resveratrol | 1457 | CSNK2A1 | 9616 | RNF7 |
| 199 | DB02709 | Resveratrol | 1457 | CSNK2A1 | 9656 | MDC1 |
| 200 | DB02709 | Resveratrol | 1457 | CSNK2A1 | 983 | CDK1 |
| 201 | DB02709 | Resveratrol | 1457 | CSNK2A1 | 994 | CDC25B |
| 202 | DB02709 | Resveratrol | 1457 | CSNK2A1 | 999 | CDH1 |
| 203 | DB02709 | Resveratrol | 4835 | NQO2 | 26003 | GORASP2 |
| 204 | DB02709 | Resveratrol | 4835 | NQO2 | 388 | RHOB |
| 205 | DB02709 | Resveratrol | 4835 | NQO2 | 5971 | RELB |
| 206 | DB02709 | Resveratrol | 4835 | NQO2 | 7157 | TP53 |
| 207 | DB02709 | Resveratrol | 5742 | PTGS1 | 4924 | NUCB1 |
| 208 | DB02709 | Resveratrol | 5743 | PTGS2 | 10987 | COPS5 |
| 209 | DB02709 | Resveratrol | 5743 | PTGS2 | 4924 | NUCB1 |
| 210 | DB02709 | Resveratrol | 5743 | PTGS2 | 50813 | COPS7A |
| 211 | DB02709 | Resveratrol | 5743 | PTGS2 | 5705 | PSMC5 |
| 212 | DB02709 | Resveratrol | 5743 | PTGS2 | 5707 | PSMD1 |
| 213 | DB02709 | Resveratrol | 5743 | PTGS2 | 7157 | TP53 |
| 214 | DB02709 | Resveratrol | 5743 | PTGS2 | 7920 | ABHD16A |
| 215 | DB02709 | Resveratrol | 5743 | PTGS2 | 8451 | CUL4A |
| 216 | DB02709 | Resveratrol | 5743 | PTGS2 | 8454 | CUL1 |
| 217 | DB02709 | Resveratrol | 5743 | PTGS2 | 8533 | COPS3 |
| 218 | DB02709 | Resveratrol | 5743 | PTGS2 | 857 | CAV1 |
| 219 | DB02709 | Resveratrol | 5743 | PTGS2 | 9978 | RBX1 |
